# Eburicoic Acid, a Triterpenoid Compound from *Antrodia camphorata,* Displays Antidiabetic and Antihyperlipidemic Effects in Palmitate-Treated C2C12 Myotubes and in High-Fat Diet-Fed Mice

**DOI:** 10.3390/ijms18112314

**Published:** 2017-11-02

**Authors:** Cheng-Hsiu Lin, Yueh-Hsiung Kuo, Chun-Ching Shih

**Affiliations:** 1 Department of Internal Medicine, Fengyuan Hospital, Ministry of Health and Welfare, Fengyuan District, Taichung City 42055, Taiwan; keny71@pchome.com.tw; 2 Department of Chinese Pharmaceutical Sciences and Chinese Medicine Resources, China Medical University, Taichung City 40402, Taiwan; kuoyh@mail.cmu.edu.tw; 3 Department of Biotechnology, Asia University, Taichung 41354, Taiwan; 4 Graduate Institute of Biotechnology and Biomedical Engineering, College of Health Science, Central Taiwan University of Science and Technology, No.666 Buzih Road, Beitun District, Taichung City 40601, Taiwan

**Keywords:** *Antrodia camphorata*, eburicoic acid, diabetes, forkhead box protein O1, fatty acid synthase, peroxisome proliferator-activated receptor α

## Abstract

This study was designed to investigate the antidiabetic and antihyperlipidemic effects and mechanisms of eburicoic acid (TRR); one component of *Antrodia camphorata* in vitro and in an animal model for 14 weeks. Expression levels of membrane glucose transporter type 4 (GLUT4); phospho-5′-adenosine monophosphate-activated protein kinase (AMPK)/total AMPK; and phospho-Akt/total Akt in insulin-resistant C2C12 myotube cells were significantly decreased by palmitate; and such decrease was prevented and restored by TRR at different concentrations. A group of control (CON) was on low-fat diet over a period of 14 weeks. Diabetic mice; after high-fat-diet (HFD) induction for 10 weeks; were randomly divided into six groups and were given once a day oral gavage doses of either TRR (at three dosage levels); fenofibrate (Feno) (at 0.25 g/kg body weight); metformin (Metf) (at 0.3 g/kg body weight); or vehicle (distilled water) (HF group) over a period of 4 weeks and still on HFD. Levels of glucose; triglyceride; free fatty acid (FFA); insulin; and leptin in blood were increased in 14-week HFD-fed mice as compared to the CON group; and the increases were prevented by TRR, Feno, or Metf as compared to the HF group. Moreover, HFD-induction displayed a decrease in circulating adiponectin levels, and the decrease was prevented by TRR, Feno, or Metf treatment. The overall effect of TRR is to decrease glucose and triglyceride levels and improved peripheral insulin sensitivity. Eburicoic acid, Feno, and Metf displayed both enhanced expression levels of phospho-AMPK and membrane expression levels of GLUT4 in the skeletal muscle of HFD-fed mice to facilitate glucose uptake with consequent enhanced hepatic expression levels of phospho-AMPK in the liver and phosphorylation of the transcription factor forkhead box protein O1 (FOXO1) but decreased messenger RNA (mRNA) of phosphenolpyruvate carboxykinase (PEPCK) to inhibit hepatic glucose production; resulting in lowered blood glucose levels. Moreover; TRR treatment increased hepatic expression levels of the peroxisome proliferator-activated receptor α (PPARα) to enhance fatty acid oxidation; but displayed a reduction in expressions of hepatic fatty acid synthase (FAS) but an increase in fatty acid oxidation PPARα coincident with a decrease in hepatic mRNA levels of sterol response element binding protein-1c (SREBP-1c); resulting in a decrease in blood triglycerides and amelioration of hepatic ballooning degeneration. Eburicoic acid-treated mice reduced adipose expression levels of lipogenic FAS and peroxisome proliferator-activated receptor γ (PPARγ) and led to decreased adipose lipid accumulation. The present findings demonstrated that TRR exhibits a beneficial therapeutic potential in the treatment of type 2 diabetes and hyperlipidemia.

## 1. Introduction

Diabetes mellitus is the core of multiple etiologies of chronic hyperglycemia. Type 2 diabetes has been proposed to display more than 90% of all diabetes mellitus patients [1]. This disease is strongly associated with obesity and insulin resistance [2] and has revealed mechanisms of insulin resistance that target the either impairs in β-cell function or insulin insensitive action at adipose tissue, skeletal muscle, or liver tissues [3].

**Figure 1:**
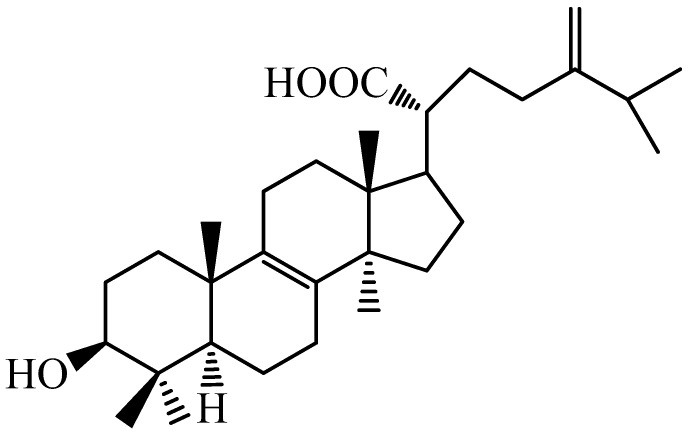
Chemical structure of eburicoic acid (TRR).

*Antrodia camphorata* (Syn. *Antrodia cinnamomea*) is a precious edible fungus endemic to Taiwan. It is rare and expensive because it grows only on the inner heartwood wall of the endemic evergreen *Cinnamomum kanehirai*. The identified compounds of *A. cinnamomea* are included as the followings: its fruiting body of *A. cinnamomea* consisted of terpenoids, zhankuic acid A, B, C, D, and E. The submerged whole broth contained 10-hydroxy-γ-dodecalactone, 11-hydroxy-γ-dodecalactone, and ergostatrien-3β-ol. The mycelium of *A. cinnamomea* contained antroquinonol and 4-acetylantroquinonol B. There are numerous biological activities of different *A. camphorata* fractions and active ingredients to be proven efficient in different animal models including cytotoxic, anti-inflammatory, and immunomodulatory activity. Our recent studies demonstrated that in different animal models, several pure compounds from *A. camphorata* including ergostatrien-3β-ol [4], dehydroeburicoic acid (TR2) [5,6], and ancin K display antidiabetic and antihyperlipidemic effects [7]. Regarding structure activity relationship (SAR), the skeleton of eburicoic acid (TRR) (Figure 1), TR2, sulphurenic acid (TR3), or dehydrosulphurenic acid (TR4) is C_31_, which is known as 24-methylenelanostane, and there is difference in structures of TR3 and TR4 existing more than –OH on C_15_’s α position on D ring.

The glucose transporter type 4 (GLUT4) plays a vital determinant of blood glucose homeostasis [8]. Skeletal muscle is the primary site of whole-body insulin-mediated glucose uptake [9]. Insulin stimulates glucose uptake in skeletal muscle by inducing net translocation of GLUT4 from the intracellular storage sites to the plasma membrane [10]. Impairment of GLUT4 expression, GLUT4 translocation and/or insulin signaling may affect insulin-stimulated glucose uptake and will result in insulin resistance and hyperglycemia [11,12]. These highlight a potential role of the improvement of GLUT4 contents and/or translocation to the plasma membrane in the treatment of diabetes mellitus.

Activation of the 5′-adenosine monophosphate protein kinase (AMPK) contributed to an increase in lipid and glucose catabolism [13]. 5′-Adenosine monophosphate protein kinase is considered to be a therapeutic target for the treatment of diabetes and dyslipidemia [14]. Since dysregulation of glucose and lipid catabolism in type 2 diabetes, AMPK activators would be promising therapies [13].

Peroxisome proliferator-activated receptor α (PPARα) plays a key role in regulation of lipid metabolism [15], and reduces circulating triglyceride (TG) concentrations via regulated numerous genes associated with fatty acids oxidation [16]. Activated AMPK reduces biosynthesis triacylglycerol in liver and then reduces concentration of TG in diabetics [17]. Moreover, AMPK activation in the liver caused an increase in fatty acid oxidation through PPARα gene expression and promoted fatty acids oxidation [18], leading to a decrease in circulating TG levels.

The present study was firstly investigated in vitro utilizing C2C12 myotube cells induced by palmitate to elucidate the potential effect and mechanism of TRR. Palmitic acid (palmitate) is the first fatty acid produced during fatty acid synthesis and is the precursor to longer fatty acids, as a result, and it makes up 21–30% of human depot fat [19]. The exact mechanism of palmitate-induced insulin resistance is still unknown, and one of the reasons is through activation of insulin–phosphatidylinositol-4,5-bisphosphate 3-kinase (PI3K)–Akt pathway [20]. Palmitate-induced cellular insulin resistance was further clarified by the reduced Akt phosphorylation, glucose uptake and GLUT4 expression [20]. The most known one is decreased glucose uptake [21]. Afterwards, this study was to look into the antidiabetic and antihyperlipidemic effects and mechanisms of TRR by the high-fat-diet (HFD)-fed mice model.

To combat type 2 diabetes, there is a need for more effective treatments. There are many animal models of type 2 diabetes; nevertheless, the development of diabetes in them is predominantly genetically determined unlike humans (including the Zucker Diabetic Fatty (ZDF) rat and *ob/ob* mouse) [22,23] or theses animal are expensive [16]. Hence, there exists a suitable and ideal animal model for type 2 to be selected for further pharmacological studies.

Here, we conduct the model of type 2 diabetes in this study by comparing the clinic anti diabetes golden drug, metformin (Meft), and the hypolipidemic drug, fenofibrate (Feno). Fenofibrate is an activator of PPARα and has been used for the management of hypertriglyceridemia for many years [24]. The model of C57BL/6J mouse that was fed a HFD is a robust and efficient model for early type 2 diabetes [25,26]. The C57BL/6J mouse is susceptible not only to HFD-induced marked increases in adipose tissue mass but also to pronounced insulin resistance, hyperlipidemia, hyperinsulinemia, hypertriglyceridemia, and hypercholesterolemia [26]. Phosphorylation of threonine 172 (Thr172) of the α subunit is essential for AMPK activity [27]. The mode of action of TRR was also investigated in a HFD-fed diabetic model. This present study examined whether TRR has its ability to translocate GLUT4 to the cell membrane to facilitate membrane glucose uptake transport and enhance phospho-AMPK or not, and targeted genes were also investigated including fatty acid oxidation peroxisome PPARα and lipogenic fatty acid synthase (FAS).

## 2. Results

### 2.1. Expression Levels of Membrane GLUT4, and Phosphorylation of AMPK and Akt In Vitro

**Figure 2:**
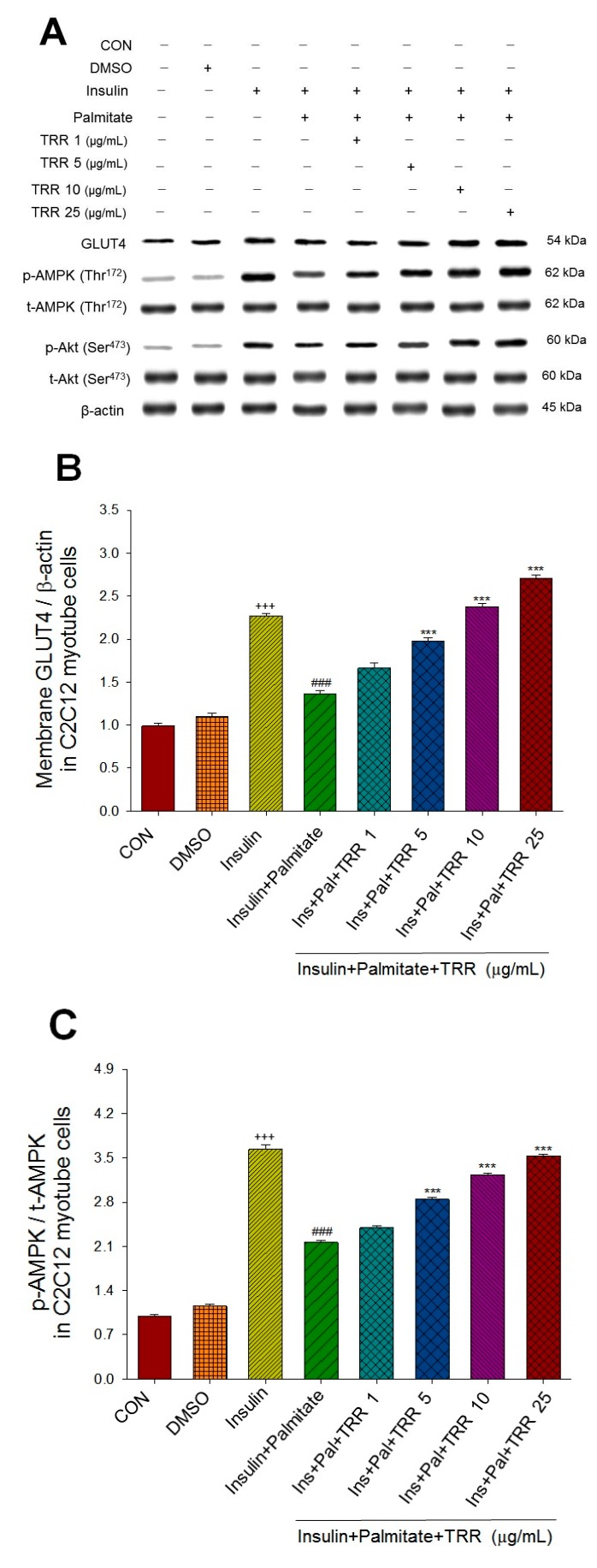

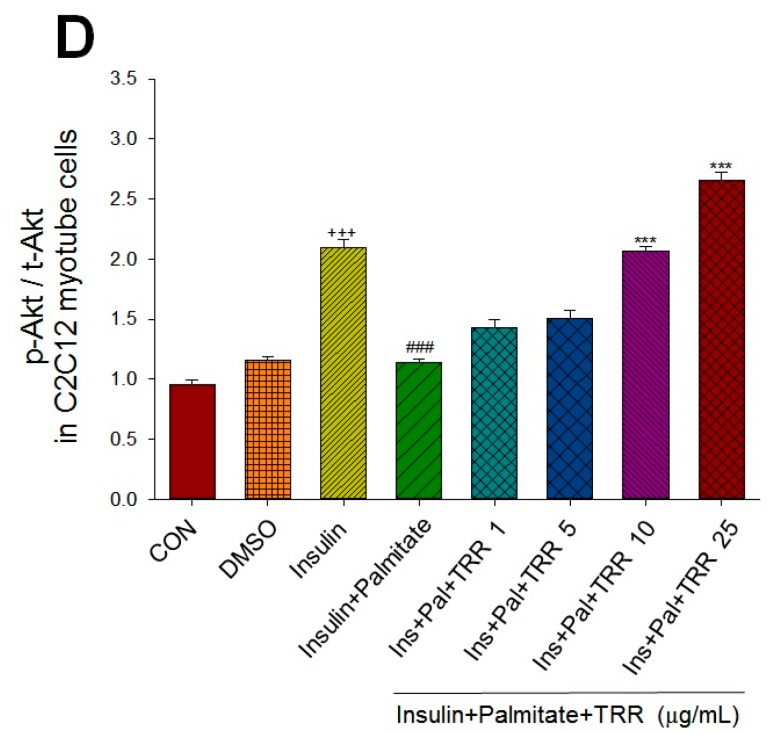
Effects of eburicoic acid (TRR) on the insulin (Ins)-stimulated expression levels of membrane glucose transporter type 4 (GLUT4), the ratio of phospho-5′-adenosine monophosphate kinase (p-AMPK) to total AMPK (t-AMPK), or phospho-Akt (p-Akt)/total Akt (t-Akt) in insulin-resistant C2C12 myotube cells induced by palmitate (Pal). The symbols “+++”, “###” and “***” represent *p* < 0.001 as respectively compared to the value of the blank control, positive control (insulin) and negative control (insulin + palmitate) using analysis of variance (ANOVA) and with Dunnett’s tests. (**A**) Representative image. (**B**–**D**) Quantification of the membrane GLUT4 expression levels, the ratio of p-AMPK to t-AMPK, or p-Akt/t-Akt expression levels. CON: Blank control; DMSO: Dimethyl sulfoxide, solvent control.

As shown in Figure 2, the negative control (as the insulin + palmitate group) exhibited insulin resistance and hyperlipidemia, resembling the symptom in the liver of HFD-induced diabetic and hyperlipidemic mice—such mice have sufficient insulin but the peripheral tissues thereof are insensitive to insulin (the insulin + palmitate group were represented as insulin resistance and hyperlipidemia). The expression levels of membrane GLUT4 and phospho-AMPK (p-AMPK)/total AMPK (t-AMPK) in the C2C12 myotubes were significantly decreased by palmitate, and such decrease was prevented and restored by 5, 10, and 25 μg/mL of TRR (Figure 2A–C). The expressions of phospho-Akt (p-Akt)/total Akt(t-Akt) levels in the C2C12 myotubes were significantly decreased by palmitate, and the decrease was prevented by 10 and 25 μg/mL of TRR (Figure 2A,D).Our results showed that TRR was not toxic to C2C12 myotubes, by the 3-(4,5-dimethylthiazol-2-yl)-2,5-diphenyltetrazolium bromide (MTT) assay.

### 2.2. Metabolic Parameters

#### 2.2.1. Oral Glucose Tolerance Test in ICR Mice

**Figure 3:**
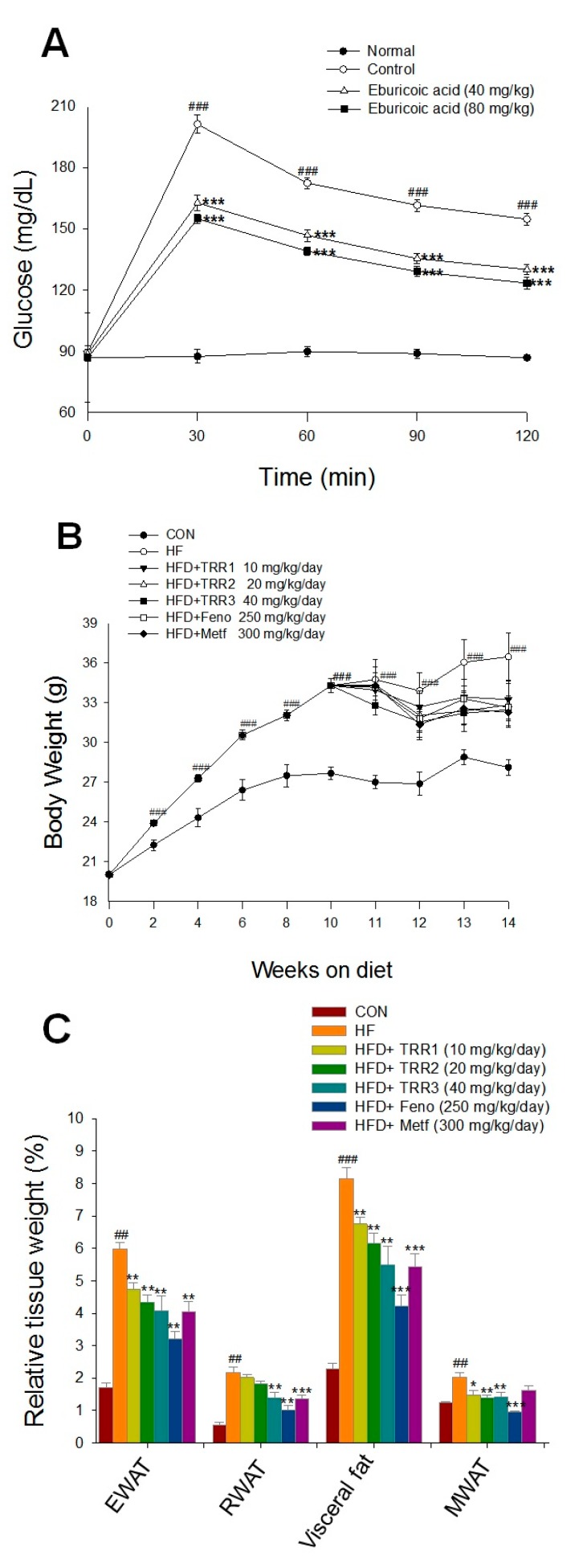

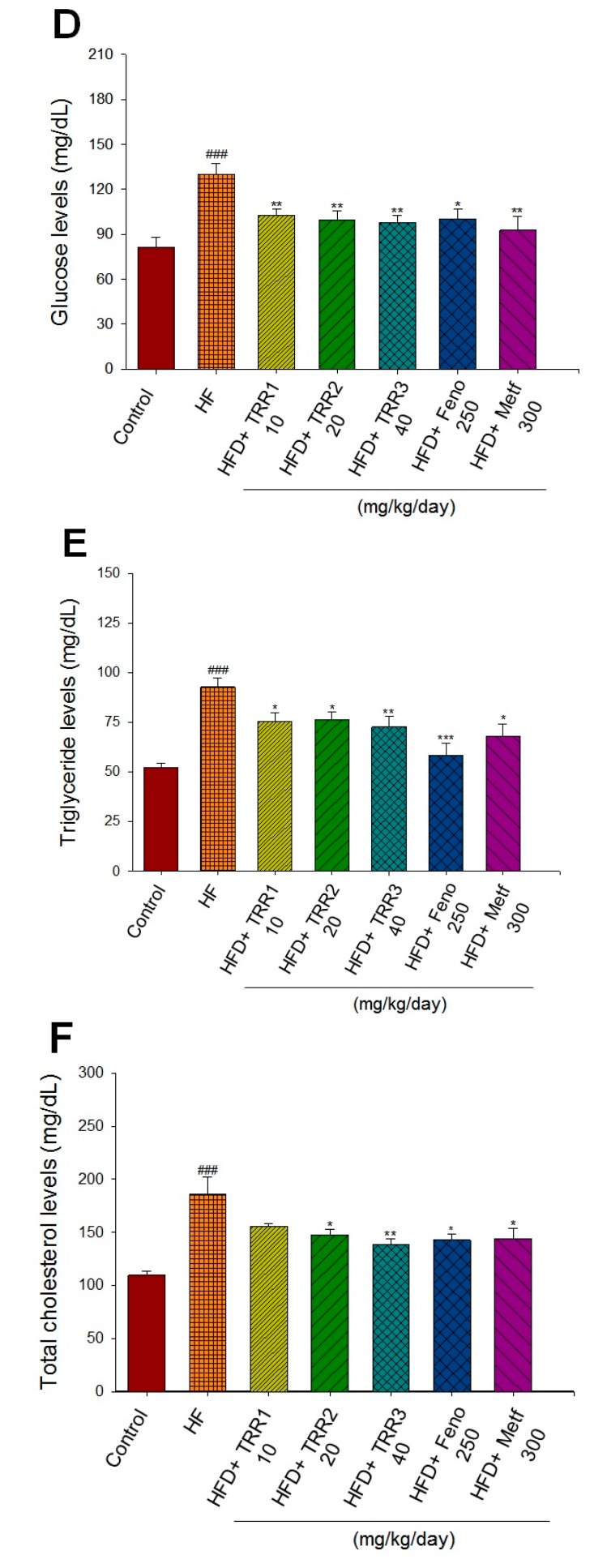

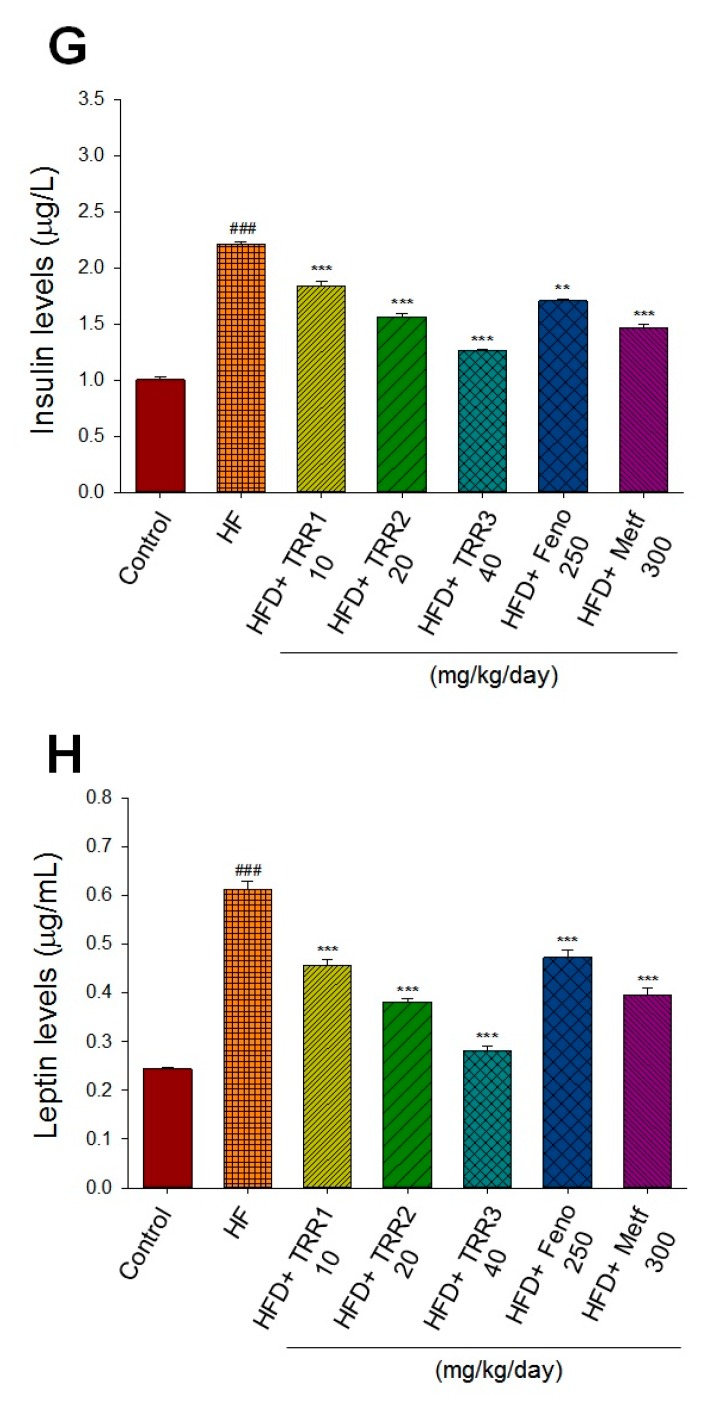

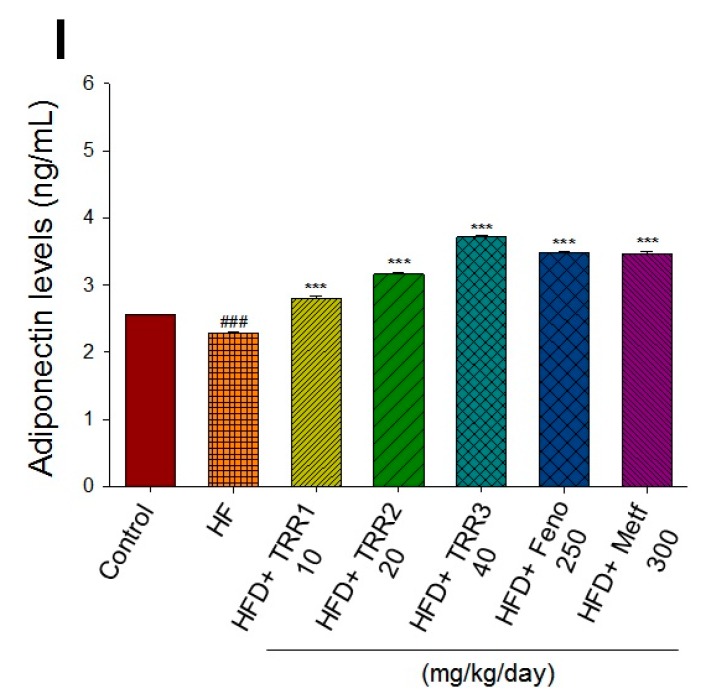
Effects of TRR including two parts. (**A**) Oral glucose tolerance (OGTT) was performed on 12 h fasted ICR mice (*n* = 5) that were allowed access to 40 and 80 mg/kg TRR or an equivalent amount of vehicle (water), which were given orally 30 min before an oral glucose load (1 g/kg body weight). The control group was given glucose, whereas the normal group was not. Blood samples were collected from the retro-orbital sinus of fasted mice at the time of the glucose administration (0) and every 30 until 120 min after glucose administration and the blood glucose level was monitored. Each point is the mean ± standard error (SE) of five separate mice. ### *p* < 0.001 compared with the control (CON) group; *** *p* < 0.001 were significantly different compared with the control group in the same time by ANOVA. (**B**–**I**) Effects of eburicoic acid (TRR) on (**B**) body weights, (**C**) relative tissue weight (%), (**D**) blood glucose levels, (**E**) blood triglycerides levels, (**F**) blood total cholesterol levels, (**G**) insulin levels, (**H**) leptin levels, and (**I**) adiponectin levels at week 14. Mice were fed with 45% high-fat diet (HFD) or low-fat diet (CON) for 14 weeks. After 14 weeks of induction, the HF mice were treated with vehicle, or eburicoic acid (TRR), or fenofibrate (Feno) or metformin (Metf) accompanied with HF diet for 4 weeks. All values are means ± SE (*n* = 9). ## *p* < 0.01, ### *p* < 0.001 compared with the control (CON) group; * *p* < 0.05, ** *p* < 0.01, and *** *p* < 0.001 compared with the high-fat-diet (HFD) plus vehicle (distilled water) (HF) group using ANOVA and with Dunnett’s tests. EWAT: Epididymal white adipose tissue; Feno: Fenofibrate (250 mg/kg body weight); Metf: Metformin (300 mg/kg body weight); MWAT: Mesenteric white adipose tissue; RWAT: Retroperioneal white adipose tissue; TRR1, TRR2, or TRR3: 10, 20, or 40 mg/kg body weight eburicoic acid, respectively; Visceral fat: EWAT + RWAT.

As shown in Figure 3A, treatment with 40 and 80 mg/kg TRR, the levels of blood glucose were significantly decreased at 30, 60, 90 and 120 min glucose-loading as compared with the control. 

#### 2.2.2. Treatments in HFD-Fed Mice

As shown in Figure 3B and Table 1, HFD-induction for 14 weeks, mice increased both body weight and body weight gain. There is no difference in final body weight between all of the TRR-, Feno-, or Metf-treated group and the HF group; TRR2-, TRR3-, Feno-, or Metf-treated mice display reduced body weight gain as compared with the HF mice. The HF mice consume (g) less than that of CON mice. TRR2-, TRR3-, or Metf-treated mice consume less food intake (Table 1). HFD-fed mice displayed an increase in relative weights of epididymal, retroperitoneal and mesenteric white adipose tissue, visceral fat, and brown adipose tissue (BAT), but a decrease in relative liver weights. TRR1-, TRR2-, TRR3-, Feno-, and Metf-treated mice significantly decreased the relative weights of epididymal white adipose tissue (EWAT), and visceral fat (represented by EWAT plus RWAT) (Figure 3C). The TRR1-, TRR2-, and TRR3-treated mice reduced the relative weights of mesenteric white adipose tissue (MWAT). The TRR3-, Feno-, and Metf-treated mice reduced the relative weights of retroperioneal white adipose tissue (RWAT). There is no difference in the relative weights of BAT, whereas Feno-treated mice significantly increased the relative weights of liver tissue (Table 1). The HFD-induced diabetic mice increased free fatty acid (FFA) levels as compared with the control (CON) mice; and TRR1-, TRR2-, TRR3-, Feno-, or Metf-treated mice display significantly reduced FFA levels as compared with vehicle-treated HF mice (Table 1).

### 2.3. Blood Glucose, Triglyceride, Total Cholesterol, Insulin, Leptin, and Adiponectin Levels

High-fat diet induction for 14 weeks displayed the significant hyperglycemia and hypertriglyceridemia (*p* < 0.001 and *p* < 0.001, respectively), and following treatment with TRR1, TRR2, TRR3, Feno, and Metf significantly lowered blood glucose and triglyceride levels as compared with the HF mice (Figure 3D,E). High-fat diet-induction for 14 weeks mice markedly increased blood total cholesterol levels (*p* < 0.001), and TRR2-, TRR3-, Feno-, and Metf-treated mice decreased blood total cholesterol (TC) levels as compared with the HF mice (Figure 3F). The HF mice displayed the significant hyperinsulinemia and hyperleptinemia (*p* < 0.001, *p* < 0.001, respectively), and TRR1-, TRR2-, TRR3-, Feno-, and Metf-treated mice significantly lowered levels of blood insulin and leptin (Figure 3G,H). The HF mice caused the hypoadiponemia (*p* < 0.001), and all of the TRR-, Feno-, and Metf-treated mice markedly increased adiponectin levels as compared with the HF mice (Figure 3I).

### 2.4. Histopathology Examination

**Figure 4:**
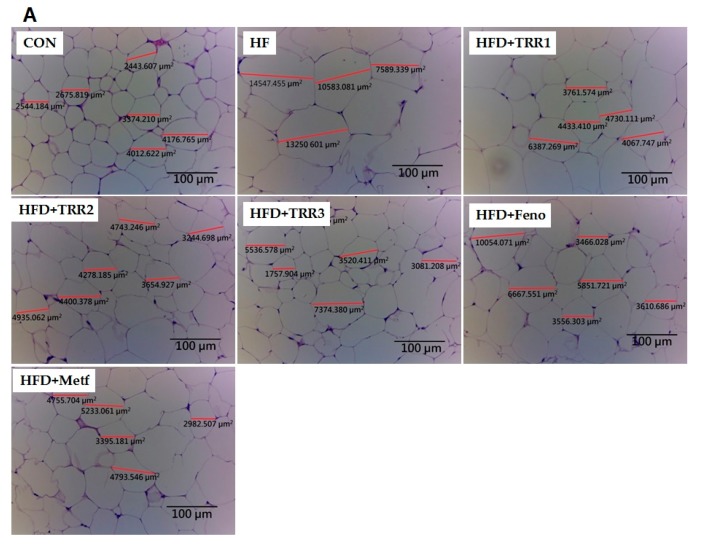

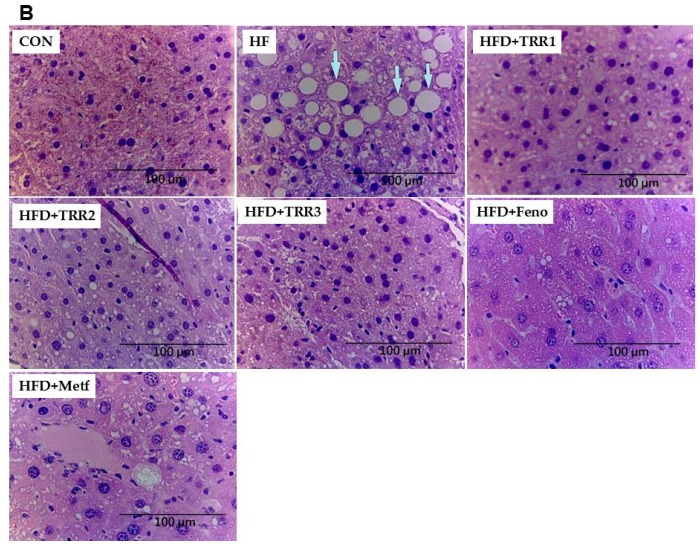
Histology of (**A**) epididymal white adipose tissue and (**B**) liver tissue of mice in the control (CON), high-fat-diet plus vehicle (distilled water) (HF), HFD + TRR1, HFD + TRR2, HFD + TRR3, HFD + fenofibrate (Feno), or HFD + metformin (Metf) groups by hematoxylin and eosin staining. Arrows indicated the hepatic ballooning degeneration. Each presented is typical and representative of nine mice, and one section per mouse. TRR1, TRR2, or TRR3: 10, 20, or 40 mg/kg body weight eburicoic acid, respectively Feno: Fenofibrate (250 mg/kg body weight); Metf: Metformin (300 mg/kg body weight).

High-fat diet caused adipocytes hypertrophy (the following data were calculated average areas: the CON mice, 3122.3 ± 373.1 μm^2^; the HF group, 9523.3 ± 788.1 μm^2^), and following treatment with TRR1 (4952.8 ± 138.1 μm^2^), TRR2 (4897.1 ± 221.1 μm^2^), TRR3 (3514.4 ± 459.7 μm^2^), Feno (5823.0 ± 288.6 μm^2^), or Metf (4682.2 ± 303.7 μm^2^) displayed less hypertrophy (Figure 4A). On the basis of a previous study [28], the designation of histological hepatocellular ballooning findings is comprised of grade 0, none; grade 1, few cells; grade 2, many cells. High-fat diet induced the ballooning of hepatocyte (mean score, 2.0 ± 0.1) as compared with the CON group (0) in liver tissue. Administration of TRR1 (0.7 ± 0.1), TRR2 (0.4 ± 0.2), TRR3 (0.4 ± 0.1), Feno (0.4 ± 0.1), or Metf (0.6 ± 0.2) decreased the ballooning as compared with the HF group (Figure 4B).

### 2.5. Hepatic Targeted Gene mRNA Levels

**Figure 5:**
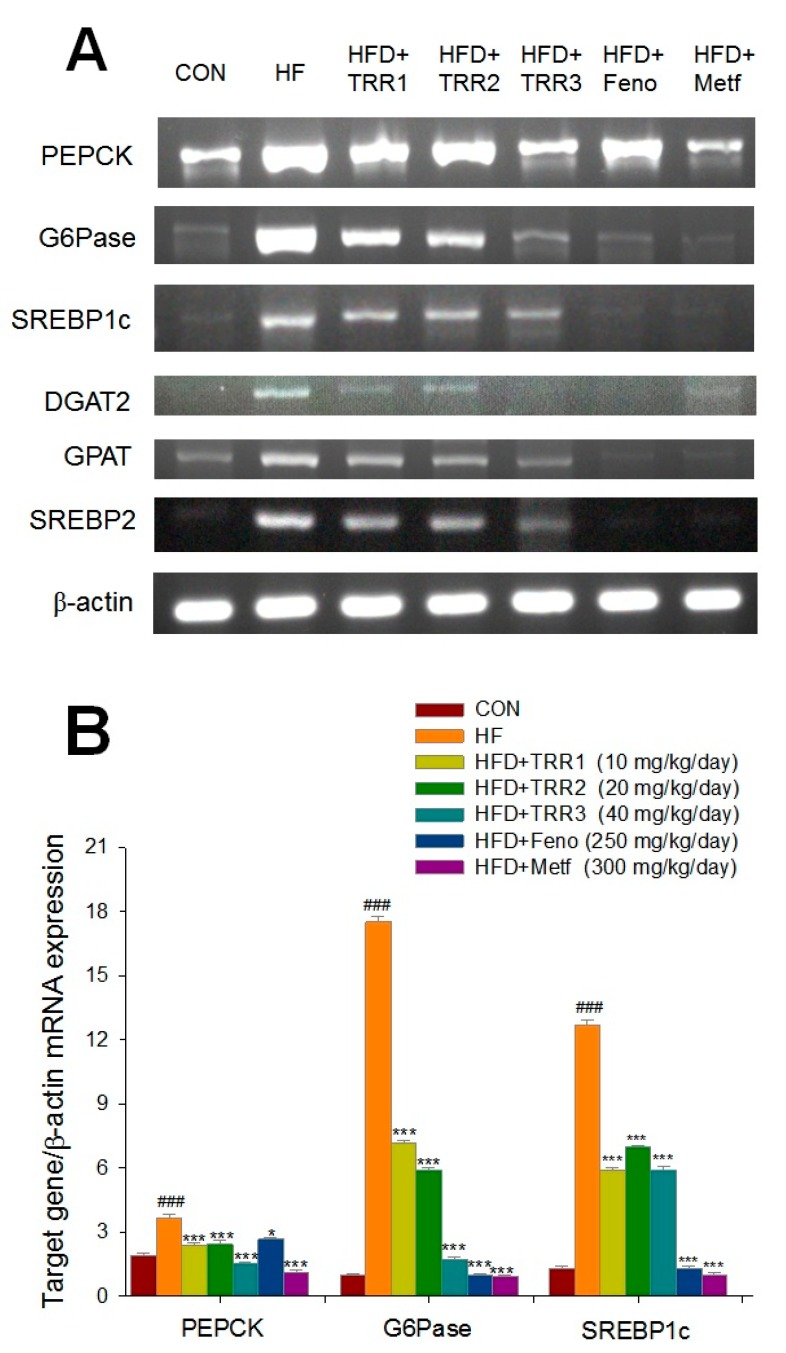

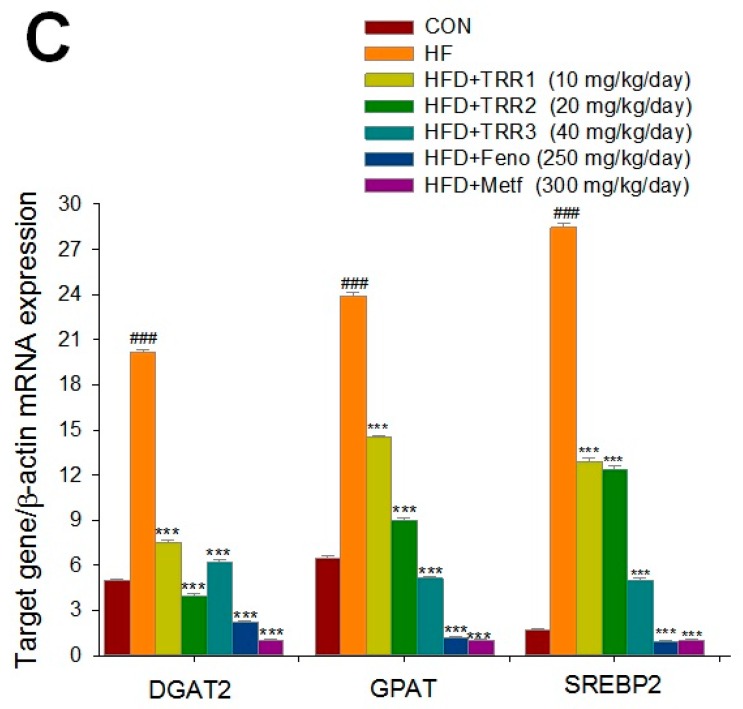
Semiquantative reverse transcription polymerase chain reaction (RT-PCR) analysis on phosphenolpyruvate carboxykinase (PEPCK), glucose-6-phosphatase (G6Pase), sterol regulatory element-binding protein 1 (SREBP1c), diacylglycerol acyltransferase 2 (DGAT2), glycerol-3-phosphate acyltransferase (GPAT), and sterol regulatory element-binding protein 2 (SREBP2) messenger RNA (mRNA) levels in liver tissue of the mice receiving eburicoic acid (TRR) by oral gavage for 4 weeks. (**A**) representative image. (**B**,**C**) Quantification of the ratio of target gene to GAPDH mRNA expression. All values are means ± SE (*n* = 9). ### *p* < 0.001 compared with the control (CON) group; * *p* < 0.05 and *** *p* < 0.001 compared with the high-fat diet plus vehicle (HF) group using ANOVA and with Dunnett’s tests. TRR1, TRR2, or TRR3: Eburicoic acid 10, 20, or 40 mg/kg body weight, respectively; Feno: Fenofibrate (250 mg/kg body weight); Metf: Metformin (300 mg/kg body weight). Total RNA (1 μg) isolated from tissue was reverse transcripted by M-MLV reverse transcriptase (M-MLV-RT), 10 μL of reverse transcriptase (RT) products were used as templates for PCR. Signals were quantitated by image analysis; each value was normalized by β-actin.

High-fat diet elicits increases in phosphenolpyruvate carboxykinase (PEPCK), glucose-6-phosphatase (G6Pase), acyl-coenzyme A:diacylglycerol acyltransferase 2 (DGAT 2), sterol regulatory element binding protein 1c (SREBP1c), glycerol-3-phosphate acyltransferase (GPAT), and sterol regulatory element-binding protein 2 (SREBP2) mRNA levels. The TRR1-, TRR2-, TRR3-, Feno-, or Metf-treated mice decreased mRNA levels of PEPCK, G6Pase, SREBP1c, DGAT2, GPAT, and SREBP2 (Figure 5).

### 2.6. Targeted Expression Levels in Different Tissues

**Figure 6:**
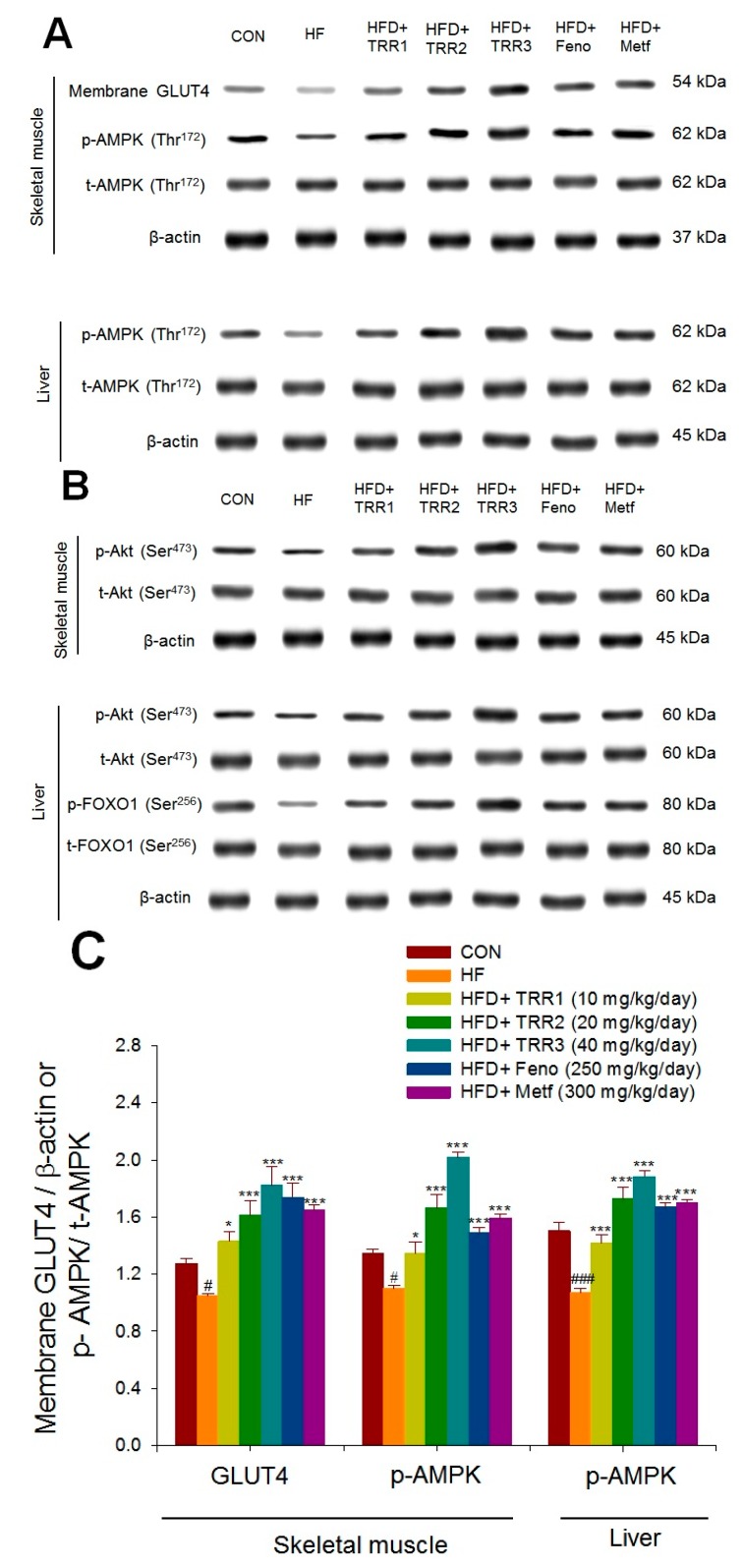

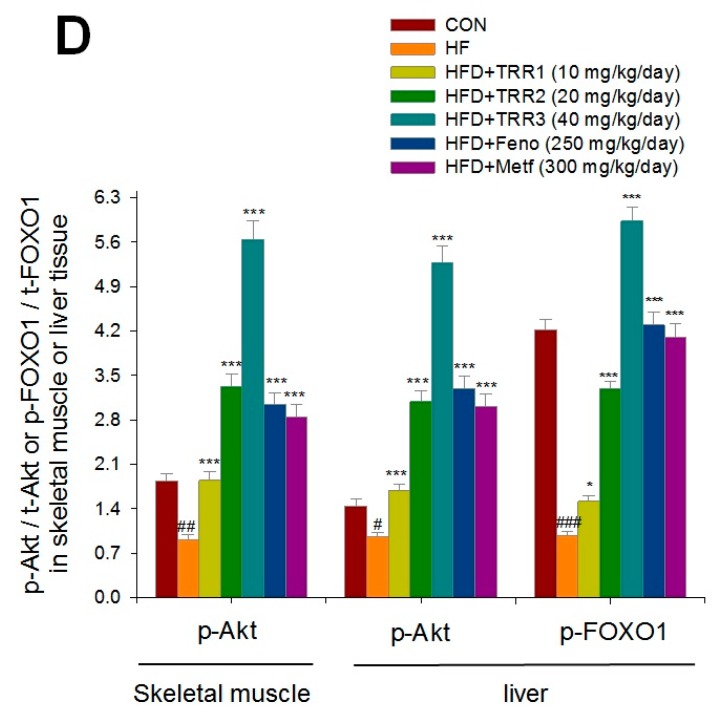
Membrane GLUT4 protein contents in skeletal muscle, or expression levels of phospho-Akt (p-Akt)/total Akt (t-Akt), phospho-AMPK (p-AMPK) (Thr172)/total AMPK (t-AMPK), or phospho-FOXO1 (p-FOXO1) (Ser256)/total FOXO1 (t-FOXO1) in liver and skeletal muscle of the mice by oral gavage eburicoic acid (TRR). (**A**,**B**) Representative images including membrane GLUT4, p-AMPK, t-AMPK, p-Akt, t-Akt, p-FOXO, and t-FOXO1 in different tissues. (**C**,**D**) Quantification of the GLUT4 expression levels, the ratio of p-AMPK to t-AMPK, or p-Akt/t-Akt expression levels (mean ± SE, *n* = 9). Protein was separated by 12% SDS–PAGE detected by Western blot. # *p* < 0.05, ## *p* < 0.01, and ### *p* < 0.001 compared with the control (CON) group; * *p* < 0.05 and *** *p* < 0.001 compared with the high-fat-diet plus vehicle (distilled water) (HF) group using ANOVA and with Dunnett’s tests. TRR1, TRR2, or TRR3: Eburicoic acid 10, 20, or 40 mg/kg body weight, respectively; Feno: Fenofibrate (250 mg/kg body weight); Metf: Metformin (300 mg/kg body weight).

High-fat diet induced decreases in expression levels of membrane GLUT4 in skeletal muscles as compared with the CON group (*p* < 0.05). TRR1-, TRR2-, TRR3-, Feno-, or Metf-treated groups enhanced membrane GLUT4 expressions as compared with the HF group (Figure 6A). High-fat diet-induced mice decreased expression levels of p-AMPK/t-AMPK both in skeletal muscle and liver tissue (*p* < 0.05 and *p* < 0.001, respectively), which were significantly increased in TRR1-, TRR2-, TRR3-, Feno-, or Metf-treated groups as compared with the HF group (Figure 6A). High-fat diet-induced mice decreased expression levels of p-Akt/t-Akt both in skeletal muscle and liver tissue (*p* < 0.01 and *p* < 0.05, respectively), which were markedly enhanced in the TRR1-, TRR2-, TRR3-, Feno-, or Metf-treated mice (Figure 6B,C). High-fat diet-induced mice decreased expression levels of p-FOXO1/t-FOXO1 in liver tissue (*p* < 0.001), and were increased in the TRR1-, TRR2-, TRR3-, Feno-, or Metf-treated mice (Figure 6B,D).

**Figure 7:**
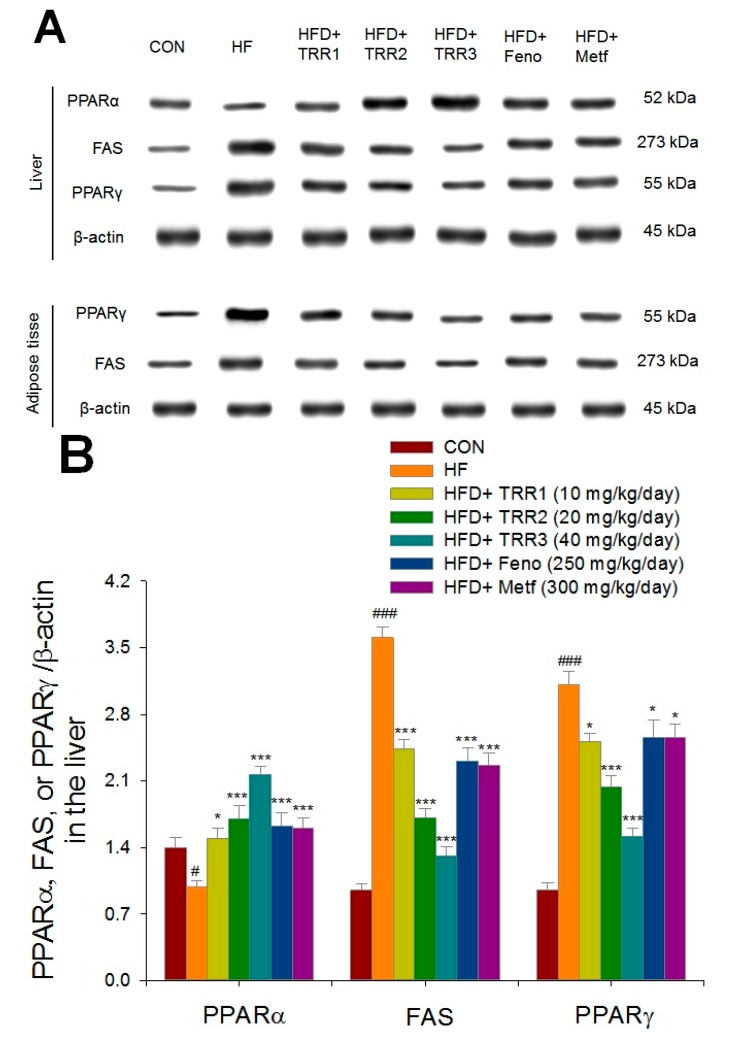

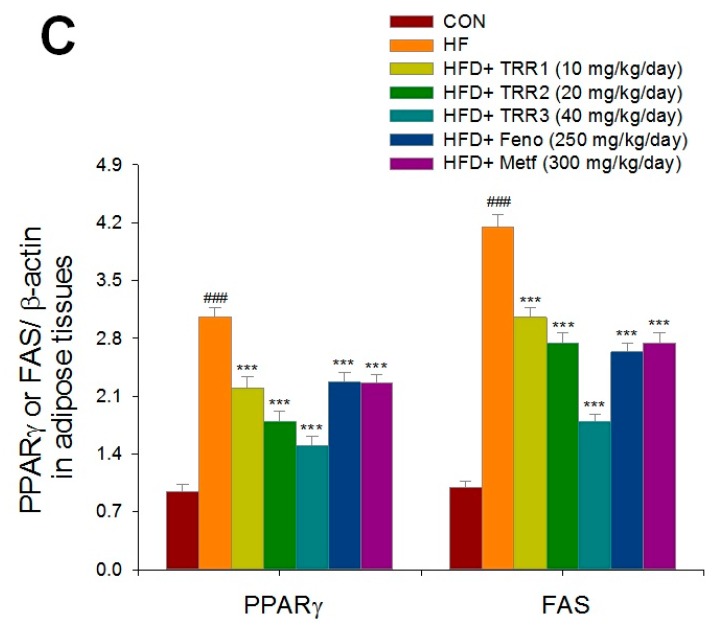
Expression levels of peroxisome proliferator-activated receptor α (PPARα), fatty acid synthase (FAS), and peroxisome proliferator-activated receptor γ (PPARγ) in the liver, and PPARγ and FAS in adipose tissue of mice by oral gavage eburicoic acid (TRR). (**A**) Representative images including PPARα, FAS, and PPARγ in different tissues. (**B**,**C**) Quantification of the expression levels of PPARα, FAS, and PPARγ in the liver and expression levels of FAS and PPARγ in adipose tissue. Protein was separated by 12% SDS–PAGE detected by Western blot. All values are means ± SE (*n* = 9). # *p* < 0.05 and ### *p* < 0.001 compared with the control (CON) group; * *p* < 0.05 and *** *p* < 0.001 compared with the HFD plus vehicle (distilled water) (HF) group using ANOVA and with Dunnett’s tests. TRR1, TRR2, or TRR3: 10, 20, or 40 mg/kg body weight eburicoic acid, respectively; Feno: Fenofibrate (250 mg/kg body weight); Metf: Metformin (300 mg/kg body weight).

High-fat diet-fed mice reduced hepatic PPARα expressions, but increased in FAS and PPARγ expressions as compared with the CON mice (Figure 7A). The TRR1-, TRR2-, TRR3-, Feno-, or Metf-treated mice show an increase in the expression levels of PPARα, but a decrease in expressions of FAS in the liver; and TRR1-, TRR2-, or TRR3-treated mice reduced the expression levels of PPARγ as compared with the HF mice (Figure 7A,B). Expression of PPARγ and FAS in adipose tissue increased in the HF group. The TRR2-, TRR3-, Feno-, or Metf-treated mice decreased expression levels of PPARγ and FAS in adipose tissue (Figure 7A,C).

## 3. Discussion

Our present results provide novel evidence indicating the antidiabetic and antihyperlipidemic effects of TRR both in vitro and in vivo. In this study, C57BL/6J mice on HFD exhibit hyperglycemia, hyperinsulinemia, hypertriglycemia, and hypercholesterolemia, which were in accordance with previous study [25,26]. Visceral adiposity is strongly linked to insulin resistance, type 2 diabetes, and dyslipidemia [29]. The amount of intra-abdominal fat is associated with the risk for development of type 2 diabetes [30]. Regardless of the site of origin, high levels of FFAs in the circulation will also impair peripheral glucose disposal [31]. The present study demonstrated that TRR treatment not only significantly lowered the levels of glucose, insulin, triglycerides, total cholesterol, and FFAs in blood, but also reduced visceral fat weights. The overall studies have demonstrated a convincing outcome of TRR to have protective effects on insulin resistance in HFD-fed diabetic mice.

Plasma levels of adiponectin are negatively associated with obesity and insulin resistance [32], and low levels of adiponectin can predict the future risk of developing type 2 diabetes [33]. In contrast, high levels of adiponectin predict enhanced insulin sensitivity of both glucose and lipid metabolism [34,35]. In this study, HFD-fed mice displayed adiposity and hypoadiponectin, but hyperleptineamia in blood. Eburicoic acid treatment enhanced circulating adiponectin levels. An increase in adiponectin levels or gene expressions has shown to be beneficial for insulin resistance [35]. Therefore, TRR could provide a therapeutic advantage to ameliorate insulin resistance. Moreover, TRR treatment improved glycemic control and with favorable lipid profiles, implying that these favorable effects of TRR with adiponectin and leptin levels confers a protective effect on insulin resistance.

The 5′-adenosine monophosphate kinase activator 5-aminoimidazole-4-carboxamide ribonucleotide (AICAR) is shown to display the activity of lowering plasma glucose and amelioration of insulin resistance in animal experiments [36,37]. This work has extended to administration of HFD-fed mice with TRR for 4 weeks, with the finding that TRR increased the expressions of membrane GLUT4 in skeletal muscles (Figure 6) and enhanced expression levels of p-AMPK both in skeletal muscles and liver tissues (Figure 6), resulting in the improvement of both glycemic control and lipid profile, establishing TRR acts as an AMPK activator and promotes membrane glucose uptake with an increase in membrane GLUT4 expressions in the maintenance of euglycemia and normal lipidemia in a mouse model of type 2 diabetes and dyslipidemia.

Skeletal muscle is the primary site of glucose uptake, disposal, and storage that accounts for approximately 75% of the entire body’s glucose uptake under insulin stimulation [38]. Insulin stimulates glucose uptake in skeletal muscle primarily by inducing net translocation of GLUT4 from the intracellular storage sites to the plasma membrane [10]. C2C12 myotube is known to be a useful model for analyzing GLUT4 translocation in skeletal muscle [39]. Thus, membrane of GLUT4 was chosen in C2C12 myotubes and skeletal muscle as the targeting to assess the antidiabetic mode of action of TRR. There are two pathways in relation to the promoted glucose uptake into skeletal muscle including the insulin-dependent mechanisms leading to Akt/protein kinase B (PKB) activation and contraction-regulated stimulation [40,41] or hypoxia-regulated AMPK activation [41,42]. Akt/PKB stimulates glucose uptake by modulating GLUT4 [43]. First, in vitro assay (Figure 2), TRR markedly increased expressions of p-Akt/t-Akt (at 10 and 25 μg/mL), and both of membrane GLUT4 and p-AMPK/t-AMPK (at 5, 10, and 25 μg/mL). We found that 5 μg/mL of TRR did not influence p-Akt but increased membrane GLUT4 expressions; moreover, 10 and 25 μg/mL of TRR may increase the expressions of p-Akt, p-AMPK, and GLUT4, suggesting that in palmitate-treated C2C12 myotube cells, TRR could stimulate convergent glucose transport activity, presuming possibly through insulin-dependent pathway or/and activation of AMPK.

The translocation of GLUT4 is mainly regulated by two independent pathways: the insulin signaling pathway and the AMPK pathway. Also, phosphorylation of AMPK is known as another major regulator of GLUT4 translocation during exercise or in response to some antidiabetic agents such as AICAR or metformin [8]. It is clear that activation of AMPK improves symptoms of impaired glucose homeostasis and insulin resistance [44,45]. The present results conducted over the expressions of p-AMPK both in skeletal muscles and liver tissues as identified distinct roles for TRR. Second, in this HFD-fed diabetic mice, TRR treatment increased membrane GLUT4 expression levels, indicating that TRR improved glucose utilization in skeletal muscles by restoring translocation GLUT4 to the plasma membrane. In HFD-fed diabetic mice, we found that administration of TRR was able to enhance the phosphorylation of AMPK, phosphorylation of Akt, and membrane GLUT4 contents in skeletal muscles to a relative level (Figure 6), suggesting that TRR could exhibit antihyperglycemic effects with convergent increased membrane GLUT4 levels, presuming possibly through an insulin-dependent pathway or/and activation of AMPK in skeletal muscles. 

In addition, since AMPK is a major cellular regulator of lipid and glucose metabolism and its activation in muscles increases glucose uptake and fatty acid oxidation [46], the biological function of TRR on AMPK activation increases its therapeutic potential as a metabolic regulator (results in lowering TG levels) and insulin sensitizer.

Both of insulin and phosphorylation of AMPK is demonstrated to inhibit mRNA and protein of PEPCK G6Pase gene transcription [47]. Insulin inhibits gluconeogenesis via Akt-dependent phosphorylation of FOXO1, in turn, to inhibition of *PEPCK* and *G6Pase* gene transcription [48]. The transcription factor FOXO1 also activates transcription of G6Pase expression, which is required for gluconeogenesis [49]. Metformin is known to activate AMPK and lowers blood glucose concentration both by inhibition of hepatic glucose production and promotion of glucose disposal in skeletal muscle [50]. In HFD-fed diabetic mice, TRR treatment increased hepatic phosphorylation of AMPK but suppressed mRNA levels of PEPCK and G6Pase. These results demonstrated that TRR partly acts as the mode of AMPK via downregulation of PEPCK and G6Pase to inhibit hepatic glucose production. Additionally, treatment with TRR significantly increased the levels of hepatic Akt phosphorylation in HFD-fed mice. The p-FOXO1 expression levels were decreased in the liver of HFD-fed mice, as was demonstrated in a previous study [51], and increased in the presence of TRR. These results suggest that TRR partially influences FOXO1 activity by suppressing the insulin pathway in mice fed a HFD. These overall results implied that TRR has the ability to improve hyperglycemia possibly partly through insulin–pAkt–p-FOXO1 pathway or/and partly via AMPK activation to inhibit glucose production in liver tissues.

This study was conducted to further evaluate the antihyperlipidemic effect and mechanisms of TRR. Activation of AMPK increased fatty acid oxidation but inhibited lipid synthesis [36,37]. Sterol regulatory element binding protein 1c (*SREBP1c*) has been shown to upregulate a variety of lipogenic genes [52]. In PPARα-deficient mice, dysregulation of SREBP-mediated lipogenic genes have been noticed [53], suggesting the role of PPARα in SREBP-mediated regulation of lipogenic genes. Acyl-coenzyme A:diacylglycerol acyltransferase 2 (*DGAT2*) is responsible for catalyzing the terminal pace of triacylglycerol synthesis, and has a central role in intracellular accumulation [54]. Acyl-coenzyme A:diacylglycerol acyltransferase 2 gene expression increases during adipogenesis, which is accompanied by an increase in TG synthesis [55]. Metformin has been shown to downregulate the SREBP1c expression, thereby decreasing the FAS expression level through AMPK activation [13]. Glycerol-3-phosphate acyltransferase catalyzes the initial step of glycerolipid synthesis, and plays a key role in the regulation of TAG synthesis; moreover, the low mRNA and protein expressions in the modulation of mitochondrial GPAT would lead to disorders of lipid metabolism [56]. In this study, administration of TRR, Feno, or Metf revealed lipid-lowering effects and increased the expression levels of PPARα in the liver with enhanced fatty acid (FA) oxidation. On the other hand, TRR, Feno, or Metf treatment decreased the mRNA levels of SREBP1c, DGAT2, and GPAT in the liver, with consequent reduced expression levels of enzymes regulating fatty acid synthesis, including decreased expressions of FAS, and inhibition of DGAT2 and GPAT, also leading to decreased FA synthesis. Our results suggest that TRR improves plasma lipid profiles by PPARα-mediated pathway and downregulating SREBP1c expressions, thereby decreasing the FAS expressions and/or through AMPK activation.

Peroxisome proliferator-activated receptor γ is known to stimulate adipogenesis and lipogenesis in adipose tissue [24], it is abundantly expressed in adipocytes and its expression is markedly induced during adipocyte differentiation [57]. The results presented here demonstrated that administration of TRR, Feno, or Metf reduced expressions of adipogenic PPARγ and FAS in adipose tissue, lead to inhibited adipose differentiation and decreased lipid accumulation in adipose tissues.

The present results show that there is no dose-dependent effect of TRR in blood glucose levels and final body weight may be due to the fact that the production of the adipocytokines is affected by more complex factors in vivo [58]. Future studies on these problems will further clarify the modes of action of TRR involved in its impact on adipocytokines. Treatment of rat adipocytes with globular domain of adiponectin was shown to increase in the glucose uptake and AMPK activation [59]. The TRR-treated mice dramatically enhanced activation of AMPK, membrane glucose uptake, and adiponectin levels, but decreased leptin levels. Minokoshi et al. [60] showed that leptin activated AMPK, which is strongly linked to the induction of gene expression, such as PPARα, the enhancement of fatty acid oxidation but suppression of hepatic lipid accumulation. It is noteworthy finding of this study that administration of TRR significantly enhanced the phosphorylation of AMPK. Based on the results of Minokoshi et al. [60], the activation of AMPK by TRR may be associated with leptin and adiponectin secretion. It is possible that TRR could directly activate AMPK, or affect secretion of leptin and adiponectin in turn to induce AMPK activation, and it remained to be clarified.

The WAT histological findings showed that TRR decreased the number of large adipocytes. The accumulated lipids in adipose tissues is demonstrated to largely derive from circulating TG [61], and the liver is a major target tissue for lipid and lipoprotein metabolism, suggesting TRR is probably mobilize fat from adipose tissue by increasing lipid catabolism in the liver due to TRR suppressed TG synthesis but enhanced catabolism in the liver. Moreover, these effects are mediated by modulation of cytokine secretion, in turn, to reduce plasma TG levels, and suppress adipogenic PPARγ and FAS expression in the visceral. The overall effect of TRR effectively reduced the size of WAT adipocytes. In conclusion, the overall of effect of TRR may be beneficial for the management of type 2 diabetes and hypertriglyceridemia.

## 4. Materials and Methods

### 4.1. Chemicals

Rabbit anti-GLUT4 (no. sc-7938) was obtained from Santa Cruz Biotech (Santa Cruz, CA, USA). Rabbit anti-phospho-AMPK (Thr172) (no. 2531) was purchased from Cell Signaling Technology (Danvers, MA, USA). Rabbit anti-PPARα (no. ab8934), and rabbit anti-PPARγ (ab45036) antibodies were purchased from Abcam Inc. (Cambridge, MA, USA). Rabbit anti-FAS (3180), rabbit anti-phospho-Akt (Ser473) (no. 4060), rabbit anti-total AMPK (Thr172) (no. 2532), rabbit anti-phospho FOXO1 (Ser256) (no. 11115), rabbit anti-total-FOXO1 (Ser256) (no. 2880), and rabbit anti-β-actin (no. 4970) antibodies were obtained from Cell Signaling Technology (Danvers, MA, USA). Secondary anti-rabbit antibodies were purchased from Jackson ImmunoRes. Lab., Inc. (West Grove, PA, USA).

### 4.2. Isolation and Determination of the Active Compound

The mycelium of *A. camphorata* (Syn. *A. cinnamomea*) was obtained from the Grape King Biotech Inc., Chung-Li City, Taiwan. Freeze-dried powders of the mycelia of *Antrodia camphorata* (3.0 kg) were extracted three times with methanol (12 L) at room temperature (4 days × 3). The methanol extract was evaporated in vacuo to yield a brown residue, which was suspended in H_2_O (1 L) and partitioned with ethyl acetate (EtOAc; 1 L × 3). The EtOAc fraction (200 g) was chromatographed on silica gel using mixtures of hexane and EtOAc of increasing polarity as eluents and further purified with high performance liquid chromatography (HPLC;Shimadzu CL 20-A, Kyoto, Japan) on a Hibar pre-packed column RT 250-10 with chloroform: EtOAc (7:1). The flow rate was 3 mL/min, and the injection volumes of samples were 100 μL. This part of the procedure was performed in accordance with prior reports [5] Eburicoic acid was isolated by HPLC on a Hibar pre-packed column RT 250-10 with chloroform: EtOAc (7:1) and a refractive index (RI) (Knauer RI detector 2400, Hegauer Weg 38, Berlin, GermanyCity, State, Country). The flow rate was 3 mL/min, and the injection volumes of samples were 100 μL [5,62]. The yields of TR1 obtained were about 0.1% (*w*/*w*). The purity of TR1 was higher than 99% [63,64].

Eburicoic acid: ^1^H NMR (300 MHz, pyridine-*d*_5_); δ 3.41 (1H, br t, *J* = 7.6 Hz, H-3), 1.00 (3H, s, H-18), 1.06 (3H, s, H-19), 2.63 (1H, td, *J* = 2.4, 10.6 Hz, H-20), 2.27 (1H, m, H-25), 1.01 (6H, d, *J* = 7.6 Hz, H-26 and H-27), 4.87 (1H, br s, H-28a), 4.91 (1H, br s, H-28b), 1.05 (3H, s, H-29), 1.22 (3H, s, H-30), 1.00 (3H, s, H-31).

### 4.3. Palmitate Solution Preparation

Palmitate was dissolved in 0.1 M NaOH to a concentration of 75 mM in a heating water bath at 70 °C under shaking. Then, the solution was diluted with 10% FFA-free bovine serum albumin (BSA) (Merck, Darmstadt, Germany)–Dulbecco’s modified Eagle’s medium (DMEM) (Gibco BRL, Bedford, Massachusetts, USA) in the heating water bath at 55 °C under shaking, followed by conducting filtration using a membrane filter having a pore size of 0.45 μm. The thus obtained palmitate solution having a concentration of 5 mM was stored at −20 °C and used within 2 weeks. Before use, the stored palmitate solution was heated to 55 °C for 15 min and then cooled to room temperature.

### 4.4. Cell Culture and Treatment

Myoblast C2C12 cells from the American Type Culture Collection (ATCC^®^, CRL-1772) were employed and cultured as a previous report [5]. Myoblast C2C12 cells were incubated in DMEM) supplemented with 10% fetal bovine serum (FBS, Hyclone, South Logan, UT, USA), 100 U/mL penicillin and 100 μg/mL streptomycin (Gibco BRL, Bedford, MA, USA) under a humidified atmosphere with culture conditions set at 37 °C and 5% CO_2_. Cells were reseeded in a culture plate at a density of 2 × 10^4^ cells/mL. Eburicoic acid was added at various concentrations 2 h prior to the experiments. After cell attachment, the cultured cells were respectively treated with the TRR for 2 h. Afterwards, the myoblast C2C12 cells were treated with the palmitate solution (in order for the cultures to have a final palmitate concentration of 0.75 mM) for 16 h, followed by performing incubation with insulin (100 nM) for 30 min. Myoblast C2C12 cells that were not treated with one of different concentrations of TRR before the addition of the palmitate solution and that were later treated with the palmitate solution and insulin served as the negative control. Myoblast C2C12 cells that were only co-cultured with insulin (without TRR and palmitate) served as a normal control (as the insulin group). Myoblast C2C12 cells that were not treated with the TRR, dimethyl sulfoxide (DMSO) (Sigma-Aldrich, St Loiuis, MO, USA), insulin, and palmitate served as a blank control (as the control group). Myoblast C2C12 cells that were co-cultured only with DMSO (without the TRR, palmitate, and insulin) served as a solvent control (as the DMSO group).

### 4.5. Detection of Expression Levels of Membrane GLUT4 and Phosphorylation of Akt (Ser473) *In Vitro*

The above treated cells were harvested and lysed. The obtained cell lysate was centrifuged, and the supernatant was resuspended to obtain a total protein sample. Assays of GLUT4 were performed within the membrane. Skeletal muscle from mice is subjected to analyses of GLUT4 protein expressions, and total membrane fraction was collected and determined by a described procedure [65,66]. Skeletal muscle was powdered under liquid nitrogen and homogenized in buffer (pH 7.4) containing 250 mmol/L sucrose, 50 mmol/L Tris, and 0.2 mmol/L edetic acid for 20 s. The homogenate was centrifuged at 9000× *g* for 10 min at 4 °C and the supernatant was reserved. The pellets were cleaned with buffer and centrifuged for three times. All three supernatants were mixed and centrifuged at 190,000× *g* for 60 min at 4 °C. The resulting pellet was resuspended in a small amount of buffer (about 0.5 mL) as a total membrane fraction [65,66]. The concentration of the total protein sample was analyzed using a bicinchoninic acid assay (BCA) assay (Pierce, Rockford, IL, USA). Equal amounts of total protein were subjected to four-fold dilution using a SDS sample buffer, and subjected to a Western blotting experiment so as to detect the expression levels of a Western blotting experiment so as to detect the expression levels of t-AMPK, p-AMPK (Thr172), t-Akt, p-Akt (Ser473), and β-actin.

### 4.6. Animals and Treatments

The experimental protocol was performed and approved by our Institutional Animal Care and Use Committee (IACUC) (104-CTUST-03 part 2; approved on 23 December 2015 and approved by 103-CTUST-08 part 1; approved on 2014/12/19); and approved by local animal ethics committee (Affidavit of Approval of Central Taiwan Institutional Animal Ethics Committee, permit # P104-I03, # P103-I10, respectively). C57BL/6J mice (4-week old) and ICR mice (4-week old) were obtained from the National Laboratory Animal Breeding and Research Center, Ministry of Science and Technology, Taipei, Taiwan. All mice were acclimated to laboratory conditions for 7 days before commencement of the experiment. The present study contained two parts: of including part 1: OGTT. The ICR mice normal mice (*n* = 5) were fasted for 12 h but were allowed access to 40, 80 mg/kg TRR or an equivalent amount of normal vehicle (water) was given orally 30 min before an oral glucose load (1 g/kg body weight). Blood samples were collected from the retro-orbital sinus of fasting mice at the time of the glucose administration (0) and every 30 min until 120 min after glucose administration to determine the levels of glucose. Part 2, the diet and dosing design, lasted for 14 weeks throughout this study. Mice were randomly divided into two groups including the CON group (the normal control group) and the experimental group. The CON group (*n* = 9) received a low-fat diet (Diet 12450B, Research Diets, Inc., New Brunswick, NJ, USA), while the experimental group was fed a 45% HFD (Diet 12451, Research Diets, Inc., New Brunswick, NJ, USA) for 10 weeks. The low-fat diet was composed of fat 10%, while HFD was composed of fat 45% (of total energy, % kcal). The HFD-treated mice (the experimental group), after 10 weeks on HFD, were then randomly divided into six groups (*n* = 54). Three groups were treated with TRR at either 10, 20, or 40 mg/kg (groups TRR1, TRR2 or TRR3, respectively). Three comparator groups were treated with similar volumes of either Feno (250 mg/kg), Metf (300 mg/kg), or distilled water (HF control group; HF). All treatments were delivered by oral gavage to mice once daily for a period of 28 days. The CON and high-fat control (HF) mice were gavaged identical volumes of vehicle. On the 98th day, food was deprived from the mice. At the end of the experiment, mice were euthanized using carbon dioxide. On the 99th day, the mice were sacrificed, and blood and tissues were collected for analysis. The livers, skeletal muscles, and WATs (including epididymal, mesenteric and retroperitoneal WAT) were excised and weighed, and then instantly stored in a freezer at −80 °C for analysis of target gene expression. Parts of organs were for gross pathological examinations. In addition to blood glucose, firstly, heparin (30 units/mL) (Sigma-Aldrich, St Loiuis, MO, USA) were added into the collecting blood tubes, and then drying of tubes, and instantly the heparin–processed tubes were used to collect blood samples. Plasma samples were collected and separated from whole blood by centrifugation at 1600× *g* for 15 min at 4 °C, and the action of all samples was completed within 30 min. The acquired supernatant was for analysis of circulating TC and TG (20~30 μL individually). Aliquots of plasma samples (>25 μL) were acquired for analysis of insulin, leptin, and adiponectin levels. The assessment of body weight was done daily (10:00 a.m.) throughout this experiment. Body weight changing, skin disease, food consumption, and the appearance of mice were monitored up to the end of the study. Body weight gain is regarded as the difference of body weight between one day and the next day. The amount of daily food intake is represented as the difference of the placed pellet food of the day between the remaining of the next day.

### 4.7. Biochemical Analysis of Blood Parameters

The blood samples (about ≈150–200 μL) were acquired from the retro-orbital sinus of mice after 10 h fasting (8:00 a.m.), and the levels of blood glucose were measured by automatic analyzer (model 1500; Sidekick Glucose Analyzer; YSI Inc., Yellow Springs, OH, USA), which is based on glucose oxidase method. The concentrations of plasma TG, TC, and FFA were measured by commercial kits (Triglycerides-E test, Cholesterol-E test, and FFA-C test, Wako Pure Chemical, Osaka, Japan). The levels of insulin, leptin, and adiponectin were measured by enzyme-linked immunosorbent assay (ELISA) by commercial kits (insulin ELISA kit, Sibayagi, Gunma, Japan and leptin ELISA kit, Morinaga, Yokohama, Japan; adiponectin ELISA kit, Crystal Chem, Inc., Suite A Downers Grove, IL, USA).

### 4.8. Histopathological Examinations

The specimens of hepatic and adipose tissues were fixed with formalin (200 g/kg) neutral buffered solution and embedded in paraffin. The sections (8 μm) were cut and stained with hematoxylin and eosin. A microscope (Leica, DM2500, Wetzlar, Germany) and Leica Digital camera (DFC-425-C, Leica) was utilized for microscopic examination and the images were taken using a Leica Digital camera (DFC-425-C, Leica) at magnification of 10 (ocular) × 20 (objective lens) for liver tissues, and 10 (ocular) × 10 (objective lens) for epididymal WAT (on the basis of capturing more than one adipocyte). Each presented image is typical and representative of nine mice. Consistent with described reported [4,5,67], images were photographed. The ballooning degeneration phenomenon is regarded as the hepatocyte death and central accumulated glycogen, leading to the nucleolus be squeezed to the other side.

### 4.9. Relative Quantization of mRNA Indicating Gene Levels and Western Blotting

These procedures of relative quantization of mRNA (the primers are described in Table 2) and immunoblots in the measurement of expression levels of skeletal muscular GLUT4, p-AMPK (Thr172)/t-AMPK (Thr172), p-Akt (Ser473)/t-Akt (Ser473), or p-FOXO1 (Ser256)/t-FOXO1 from muscles or livers of mice were performed as previous procedures elsewhere [4,5,65,66]. The expression levels of PPARα, FAS, and PPARγ were performed from the liver tissue, and expressions of PPARγ and FAS from the adipose tissue of mice. Skeletal muscles from mice were subjected to examine expression levels of membrane GLUT4. Total membrane fraction was measured; and the expression levels of GLUT4, p-AMPK, t-AMPK, p-Akt, and t-Akt were evaluated by Western blotting, as was demonstrated in previous studies [4,5,67].

### 4.10. Statistical Analysis

Results were represented by means and standard error. Comparisons among groups were performed using analysis of variance (ANOVA), and coupled with Dunnett’s tests. The *p*-values less than 0.05 were regarded as statistically significant differences.

## 5. Conclusions

**Figure 8:**
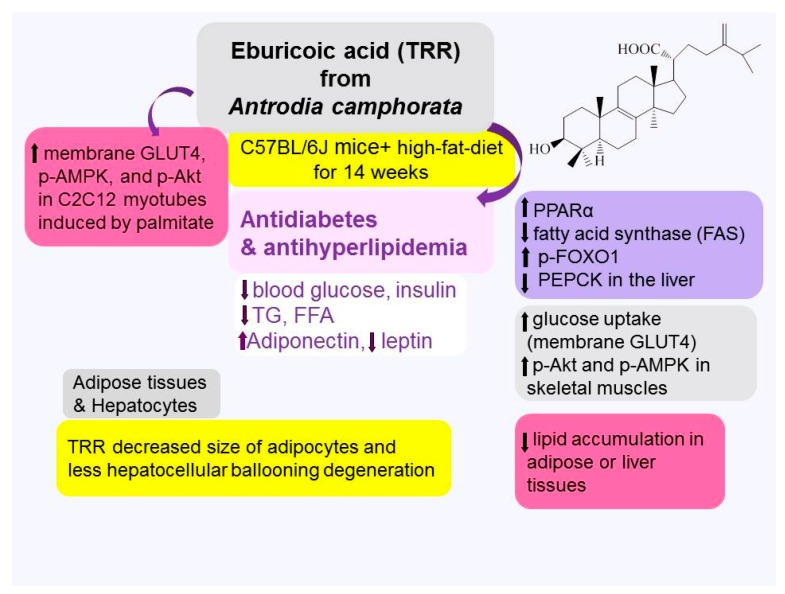
A proposed mechanism for TRR to improve diabetes and hyperlipidemia.

In conclusion, the present study demonstrated that expression levels of membrane GLUT4, p-AMPK/t-AMPK, and p-Akt/t-Akt in insulin-resistant C2C12 myotube cells were significantly decreased by palmitate, and such decrease was prevented and restored by TRR at different concentrations (see Figure 8). On the other hand, TRR displayed both antidiabetic and hypolipidemic properties in HFD-fed mice. Eburicoic acid, Feno, and Metf displayed both enhanced expression levels of p-AMPK and membrane expression levels of GLUT4 in the skeletal muscle to facilitate glucose uptake with consequent enhanced expressions of p-AMPK in the liver and increased hepatic expression levels of p-FOXO1 and suppressed mRNA levels of PEPCK and G6Pase to inhibit hepatic glucose production, contributed to TRR’s glucose-lowering effects. Eburicoic acid enhanced hepatic AMPK activation with increased SREBP1c mRNA levels and expression levels of FAS to lower hepatic triglyceride synthesis, while increased expression levels of PPARα to enhance fatty acid oxidation, thus leading to lowered circulating triglycerides levels. The overall of effect of TRR may be beneficial for the management of type 2 diabetes and hypertriglyceridemia.

## Figures and Tables

**Table 1:** Effects of TRR on relative tissue weight, food intake, and blood parameters in HFD-fed mice.

Parameter	CON	HF	HFD + TRR1	HFD + TRR2	HFD + TRR3	HFD + Feno	HFD + Metf
Dose (mg/kg/day)	0	0	10	20	40	250	300
Liver (%) ^a^	4.422 ± 0.296	3.485 ± 0.095 ^##^	3.865 ± 0.125	3.910 ± 0.164	3.661 ± 0.100 ^#^	6.512±0.258 ^###,^***	3.525 ± 0.182 ^##^
Spleen (%) ^a^	0.329 ± 0.036	0.262 ± 0.025	0.240 ± 0.007	0.252 ± 0.010	0.245 ± 0.015	0.293± 0.023	0.250 ± 0.014
BAT (%) ^a^	0.518 ± 0.032	0.946 ±0.072 ^##^	0.850 ± 0.053 ^#^	0.747 ± 0.056	0.972 ± 0.107 ^##^	0.579 ± 0.056	0.849 ± 0.144 ^#^
final body weight (g)	28.11 ± 0.58	36.48 ± 1.81 ^###^	33.25 ± 1.47 ^#^	32.84 ± 1.60 ^#^	32.48 ± 0.87 ^###^	32.65 ± 0.89 ^##^	32.27 ± 1.11 ^##^
Weight gain (g)	0.45 ± 0.17	1.53 ± 0.39 ^#^	0.79 ± 0.47	−2.23±0.29 ^###,^***	−2.08±0.78 ^###,^***	−1.83 ± 1.16 ^###,^**	−1.98 ±0.42 ^###,^***
food intake (g/day/mouse)	3.99 ± 0.03	2.71 ±0.03 ^###^	2.69 ± 0.08 ^##^	2.50 ± 0.03 ^###,^**	2.40 ± 0.03 ^###,^**	2.66 ± 0.16	2.31 ± 0.04 ^###,^***
FFA (meq/L)	4.032 ± 0.130	5.322 ± 0.191 ^###^	4.360 ± 0.159 ***	4.034 ± 0.122 ***	3.037 ±0.167 ***	1.938 ± 0.148 **	2.119 ± 0.191 ***

All values are means ± SE (*n* = 9). ^#^ *p* < 0.05, ^##^ *p* < 0.01, and ^###^ *p* < 0.001 compared with the control (CON) group; ** *p* <0.01, and *** *p* < 0.001 compared with the high-fat diet (HFD) plus vehicle (distilled water) (HF) group using ANOVA and with Dunnett’s tests. BAT: brown adipose tissue; Feno: Fenofibrate (250 mg/kg body weight); FFA: plasm free fatty acid; Metf: Metformin (300 mg/kg body weight); TRR1, TRR2, or TRR3: 10, 20, or 40 mg/kg body weight eburicoic acid, respectively. ^a^ Relative tissue weight.

**Table 2:** Primers used in this study.

Gene	Accession no.	Primers	PCR Product (bp)	Annealing Temperature (° C)
*PEPCK*	NM_011044.2	F: 5′-CTACAACTTCGGCAAATACC-3′	330	51
		R: 5′-TCCAGATACCTGTCGATCTC-3′		
*G6Pase*	NM_008061.3	F: 5′-GAACAACTAAAGCCTCTGAAAC-3′	350	50
		R: 5′-TTGCTCGATACATAAAACACTC-3′		
*SREBP1c*	NM_011480	F: 5′-GGCTGTTGTCTACCATAAGC-3′	219	48
		R: 5′-AGGAAGAAACGTGTCAAGAA-3′		
*DGAT2*	BC054791.1	F: 5′-CTTGTGACCCTACTACATCC-3′	332	51
		R: 5′-TCATAGCAGAACCTTAATCC-3′		
*GPAT*	BC019201.1	F: 5′-CAGTCCTGAATAAGAGGT-3′	441	48
		R: 5′-TGGACAAAGATGGCAGCAGA-3′		
*SREBP2*	AF289715.2	F: 5′-ATATCATTGAAAAGCGCTAC-3′	256	48
		R: 5′-AGCTCAGTAACAGTCCGCCTAGA-3′		
*β-actin*	NM_007392	F: 5′-TCTCCACCTTCCAGCAGATGT-3′	99	55
		R: 5′-AGCTCAGTAACAGTCCGCCTAGA-3′		

F: Forward; R: Reverse.
